# Screening for obstructive sleep apnea in an Afro-Caribbean Population: Diagnostic performance of common questionnaires compared with respiratory polygraphy — The MASOS Study A prospective, cross-sectional, descriptive, and analytical study

**DOI:** 10.1007/s11325-026-03619-w

**Published:** 2026-03-18

**Authors:** Moustapha Agossou, William Garnero, Bérénice Awanou, Mathilde Andreu, Nelly Ahouansou, Mathilde Provost, Marion Dufeal, Astrid Monfort-Brafine, Cédric Fagour, Mathieu Nacher, Moustapha Dramé

**Affiliations:** 1 Department of Respiratory Medicine, Centre Hospitaller Universitaire de Martinique, Route de Chateauboeuf, Fort-de-France, 97261 Martinique; 2 EpiCliV Research Unit, CHU de Martinique, Fort-de-France, Martinique; 3 Université des Antilles, Pointe-A-Pitre, Guadeloupe; 4 Department of Cardiology, Centre Hospitaller Universitaire de Martinique, Fort de France, Martinique; 5 Department of Endocrinology-Diabetology, Centre Hospitaller Universitaire de Martinique, Fort de France, Martinique; 6 Centre d’investigation clinique Antilles-Guyane, Inserm CIC1424, Centre hospitalier de Cayenne, Cayenne, French Guiana; 7 EA3593 Écosystèmes amazoniens et pathologie tropicale (EPaT), Université de Guyane, Cayenne, French Guiana; 8 Department of Clinical Research and Innovation, Centre Hospitaller Universitaire de Martinique, Fort de France, Martinique

**Keywords:** Obstructive sleep apnea hypopnea syndrome, Epworth sleepiness Scale, Berlin questionnaire, NoSAS, STOP BANG, No Apnea questionnaire, GOAL questionnaire

## Abstract

**Background:**

Obstructive sleep apnea hypopnea syndrome (OSAHS) is a major public health issue. Its prevalence is likely underestimated in many parts of the world, and experts project a substantial future increase. In settings with limited access to diagnostic tools, clinical screening questionnaires may improve the efficiency of patient management. This study aimed to evaluate the diagnostic performance of the main OSAHS screening questionnaires in an Afro-Caribbean population.

**Methods:**

We conducted a cross-sectional, descriptive, and analytical study, in a high-risk population of patients referred to the Sleep and Home Respiratory Assistance Unit of the Centre hospitalier universitaire de Martinique, using home respiratory polygraphy as the reference standard.

**Results:**

Among the 134 included patients, 60 (37.3%) were men. The mean age was 57 ± 14.9 years, the mean BMI was 30.5 ± 7.5 kg/m², and the median apnea–hypopnea index (AHI) was 11.5 [6.3–25.4]. The most frequent comorbidities were hypertension (45%), allergies (29%), asthma (25%), and diabetes (19%).

For detecting OSAHS (AHI ≥ 5), the area under the curve (AUC) was above 0.7 for the NoSAS, No-Apnea, STOP-BANG (moderate risk) and GOAL questionnaires, while the Berlin Questionnaire had an AUC of 0.65. The poorest performance was observed for the Epworth Sleepiness Scale (AUC = 0.55).

For moderate (AHI ≥ 15) and severe OSAHS (AHI ≥ 30), No-Apnea and NoSAS achieved AUCs above 0.70, whereas GOAL and STOP-BANG showed 0.67 and 0.63, respectively.

Threshold optimization using the Youden index identified higher cut-offs than standard values for some scores: No-Apnea = 6 (vs. 4), NoSAS = 10 (vs. 8), and GOAL = 3 (vs. 2).

A model combining age, BMI, and neck circumference achieved an AUC of 0.81.

**Conclusion:**

In an Afro-Caribbean population, NoSAS and No-Apnea were the most effective screening questionnaires in this referred high-risk population for OSAHS. Based on a multivariable model combining age, BMI, and neck circumference, we developed the MASOS score (Age ≥ 70 years + 2; BMI ≥ 30 kg/m² +1; neck circumference ≥ 39 cm + 1; threshold ≥ 2), which showed a higher discriminative performance (AUC = 0.79) but still requires validation.

## Introduction

Obstructive sleep apnea hypopnea syndrome (OSAHS) is a chronic respiratory disorder characterized by recurrent nocturnal breathing pauses caused by partial or complete obstruction of the upper airway. It is defined by an apnea–hypopnea index (AHI) ≥ 5 events per hour of sleep, and classified as mild (5–14/h), moderate (15–29/h), or severe (≥ 30/h) [1]. Experts estimate that the prevalence of OSAHS in the United States will increase by 34.7% by 2050, from 34.3% to 46.2%, particularly among women [2].

OSAHS is a major public health concern. It is associated with numerous cardiovascular (hypertension, myocardial infarction, atrial fibrillation), metabolic (obesity, diabetes, dyslipidemia), and neuropsychiatric (cognitive impairment, daytime sleepiness, irritability) complications [3–5]. Yet despite its high prevalence, it remains largely underdiagnosed due to limited access to specialized investigations and a lack of awareness among healthcare professionals [6].

The diagnostic gold standard is polysomnography, but this examination is often unavailable or difficult to access. Respiratory polygraphy is more widely used in routine practice, especially in ambulatory settings, but remains difficult to implement on a large scale, particularly in resource-limited areas.

To address this challenge, several screening scores have been developed to estimate the risk of OSAHS. These questionnaires are composed of objective data (BMI, age, sex) for No-Apnea and GOAL, subjective symptoms, mainly daytime sleepiness, for the Epworth Sleepiness Scale (ESS), and both for Berlin Questionnaire, STOP-BANG and NoSAS [7–12]. These questionnaires combine objective data (BMI, age, sex) and subjective data (snoring, daytime sleepiness) to estimate the risk of OSAHS. Some have shown good diagnostic performance [13], but they are still underused in primary care, partly due to a lack of large-scale validation and adaptation to specific populations.

This is particularly the case in Martinique, a French overseas department in the Caribbean, whose population has specific genetic, cultural, and environmental characteristics. Studies have reported higher prevalence of OSAHS risk factors in people of African descent, such as abdominal obesity, hypertension, insulin resistance, and distinctive craniofacial morphology [14–17]. However, local data on OSAHS prevalence and screening methods remain scarce. A 2015 report from Santé Publique France estimated the prevalence of severe OSAHS at only 2.1% in Martinique, deducted from the total number of patients fitted with devices, which is therefore surely an underestimation [18].

Access to specialized diagnostic tests is also hindered by unequal healthcare access, a shortage of trained staff, and the lack of locally validated screening tools. The performance of screening questionnaires may vary across sociocultural and linguistic contexts, particularly for patient-reported outcome (PRO) measures, as differences in daily activities, symptom perception, and item interpretation can affect measurement equivalence. This issue is especially relevant in culturally distinct settings such as the French West Indies. Moreover, several studies in United States African American populations have shown that tools such as the Berlin and STOP-BANG questionnaires have reduced performance (low sensitivity or very low specificity) [19], and require threshold adjustments. Predictive models developed specifically for Black populations, such as the one from the Jackson Heart Sleep Study, have been proposed to improve diagnostic accuracy [20]. These findings support the need to evaluate screening tools in the Afro-Caribbean population.

The objective of this study was therefore to assess the diagnostic performance of the main OSAHS screening questionnaires compared with respiratory polygraphy, and to identify clinical factors associated with OSAHS in this population.

## Materials and methods

### Study design

We conducted a prospective, cross-sectional, descriptive, and analytical study to evaluate the diagnostic performance of several obstructive sleep apnea hypopnea syndrome (OSAHS) screening tools compared with respiratory polygraphy.

### Study population and inclusion criteria

Patients were consecutively recruited from those referred to the Sleep and Home Respiratory Assistance Unit of the Centre Hospitalier Universitaire de Martinique, Department of Pulmonology, for suspected OSAHS between October 2023 and January 2025, until the required sample size was reached.

Inclusion criteria were:

- Age ≥ 18 years.
- Referral by a healthcare professional for suspected OSAHS.

Non-inclusion criteria were:

- Inability to understand or complete the questionnaire (partial assistance was allowed).
- Prior treatment for OSAHS.

### Sample size calculation

Assuming a type I error (α) of 5%, a power of 80%, a reference theoretical prevalence of 30–50%, and an expected prevalence of 70% (in this higher-risk referred population), the number of subjects required was estimated at 80 for score evaluation and 130 for association analyses. The sample size was increased by 20% to account for potential missing data. The final sample size (*n* = 134) met the a priori target for association analyses and exceeded the minimum required for questionnaire performance evaluation; however, the study was not powered for extensive subgroup or internal validation analyses.

### Questionnaires

All included patients completed a paper questionnaire that collected their medical history, treatments, anthropometric measurements, and responses to six OSAHS screening tools: Epworth Sleepiness Scale, Berlin Questionnaire, STOP-BANG, NoSAS, GOAL, and No-Apnea.

Missing questionnaire items were retrieved from medical records if the patient had been hospitalized within the previous three months. When items remained missing after medical-record review, questionnaires with incomplete data were excluded from score calculation.

### Respiratory polygraphy

All patients underwent overnight home-based respiratory polygraphy for a minimum of 6 h, including:

- oxygen saturation monitoring by oximetry.
- nasal–oral airflow recording.
- snoring quantification using tracheal sound recordings.
- respiratory effort measurement using thoracic and abdominal belts.
- body position analysis.

All polygraphy recordings were manually reviewed and scored according to current international recommendations by experienced clinicians from the sleep unit. Recordings with insufficient duration or poor signal quality were excluded to limit misclassification.

Polysomnography was not available in our unit before May 2024 and has remained available only in limited capacity since then. Therefore, respiratory polygraphy was used as the reference standard for defining OSAHS cases. Before May 2024, patients requiring polysomnography were referred to other sleep laboratories, where waiting times often exceeded 12 months.

### Exclusion criteria

Patients who completed the questionnaires but did not undergo respiratory polygraphy (*n* = 13) and those with invalid polygraphy (insufficient recording time (*n* = 21), poor signal quality (*n* = 1), or missing data (*n* = 2) were excluded. A total of 134 patients remained out of the 171 initially included.

### Statistical analysis

Data were entered in Microsoft Excel and analyzed using Python.

For diagnostic performance, we calculated sensitivity, specificity, and area under the curve (AUC) for each score, and assessed diagnostic concordance using observed agreement and Cohen’s kappa.

AUCs were tested with the Mann–Whitney U test and compared using the DeLong test.

For association analyses, univariate analyses included Student’s t-tests for normally distributed quantitative variables, Mann–Whitney U tests for non-normal quantitative variables, and odds ratios using Fisher’s exact test for binary variables. Multivariate analyses were performed for anthropometric variables and comorbidities.

Missing data were handled by complete-case analysis (listwise deletion). All variables were also evaluated with AUC to compare their discriminative performance.

### Ethical considerations

The study was conducted in accordance with the World Medical Association Declaration of Helsinki and was approved by the Institutional Review Board of the Centre Hospitalier Universitaire de Martinique (approval number 2023/032).

In accordance with French regulations, all participants received written information and provided informed consent to participate.

## Results

Among the 134 included patients, the mean age of 57 ± 14.9 years; 60 patients (37.3%) were men. The mean BMI was 30.5 ± 7.5 kg/m², and the median apnea–hypopnea index (AHI) was 11.5 [6.3–25.4]. The most frequent comorbidities were hypertension (45%), allergies (29%), asthma (25%), and diabetes (19%).

The general characteristics of the study population are presented in Table 1.

**Fig. 1:**
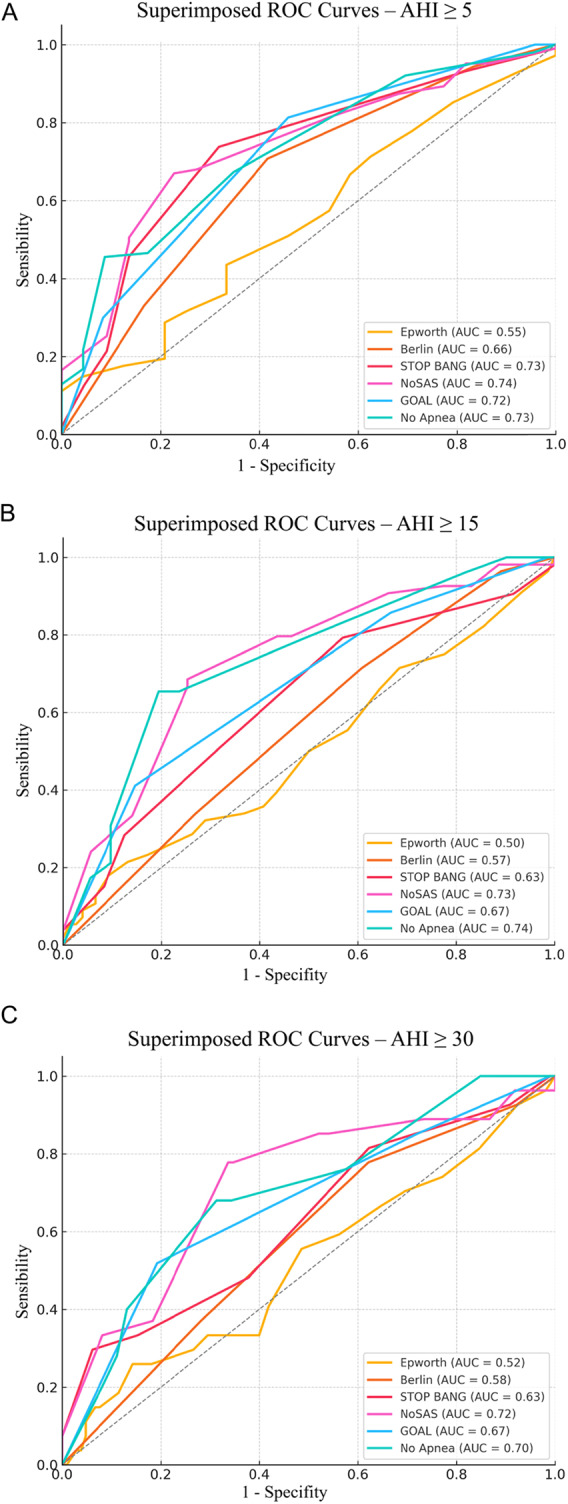
ROC curves of the different screening questionnaires according to OSAHS severity

For detecting obstructive sleep apnea hypopnea syndrome (OSAHS) (AHI ≥ 5), the NoSAS, No-Apnea, STOP-BANG, and GOAL questionnaires all showed area under the curve (AUC) values above 0.7, whereas the Berlin Questionnaire had an AUC of 0.65. The poorest performance was observed for the Epworth Sleepiness Scale (AUC = 0.55) (Fig. 1).

**Table 1:** Clinical and anthropometric characteristics of the study population

Characteristics	
AHI median [interquartile]	11.5 [6.3–25.4]
Age (year)	57.0 ± 14.9
Weight (kg)	88.1 ± 23.6
Height (cm)	169.9 ± 9.9
BMI (kg/m^2^)	30.5 ± 7.5
Neck circumference (cm)	39.5 ± 4.3
Waist circumference (cm)	106.0 ± 16.5
Male sex	50 (37.3%)
Hypertension n(%)	60 (44.8%)
Diabetes n(%)	25 (18.7%)
Dyslipidemia n(%)	11 (8.2%)
Allergies n(%)	39 (29.1%)
Rhinitis n(%)	13 (9.7%)
Sinusitis n(%)	16 (11.9%)
Asthma n(%)	33 (24.8%)

Median [Q1; Q3], Mean ± SD, N (%)

For moderate (AHI ≥ 15) and severe OSAHS (AHI ≥ 30), No-Apnea and NoSAS achieved AUCs above 0.70, while GOAL and STOP-BANG showed 0.67 and 0.63, respectively. The Epworth and Berlin questionnaires consistently demonstrated poor performance (Table 2).

**Table 2:** Agreement, sensitivity, specificity, and AUC of the different screening questionnaires for mild, moderate, and severe OSAHS

	Se	Sp	PPV	NPV	AUC	95% CI	Agreement	Kappa
AHI ≥ 5								
Epworth ≥ 16	0.15	0.96	0.83	0.19	0.55	[0.43; 0.67]	0.30	0.04
Berlin	0.71	0.58	0.88	0.31	0.66*	[0.54; 0.78]	0.68	0.22
STOP-BANG ≥ 3	0.74	0.68	0.92	0.36	0.73**	[0.61; 0.84]	0.73	0.31
NoSAS	0.68	0.73	0.32	0.33	0.74**	[0.62; 0.84]	0.69	0.27
GOAL	0.81	0.54	0.89	0.39	0.72**	[0.60; 0.81]	0.76	0.31
No-Apnea	0.67	0.65	0.89	0.31	0.73**	[0.62; 0.83]	0.67	0.23
AHI ≥ 15								
Epworth ≥ 16	0.18	0.91	0.59	0.60	0.50_	[0.41; 0.61]	0.60	0.10
Berlin	0.71	0.39	0.47	0.64	0.57	[0.47; 0.66]	0.53	0.10
STOP-BANG ≥ 3	0.79	0.43	0.51	0.74	0.63*	[0.53; 0.72]	0.58	0.21
NoSAS	0.80	0.54	0.57	0.78	0.73**	[0.64; 0.82]	0.65	0.32
GOAL	0.86	0.33	0.49	0.76	0.67**	[0.58; 0.75]	0.56	0.17
No-Apnea	0.79	0.51	0.54	0.77	0.74**	[0.64; 0.82]	0.63	0.28
AHI ≥ 30								
Epworth ≥ 16	0.19	0.89	0.29	0.81	0.52	[0.39; 0.64]	0.74	0.08
Berlin	0.78	0.38	0.25	0.87	0.58	[0.46; 0.69]	0.46	0.09
STOP-BANG ≥ 3	0.81	0.38	0.27	0.88	0.63*	[0.50; 0.74]	0.47	0.11
NoSAS	0.85	0.46	0.30	0.92	0.72**	[0.60; 0.82]	0.54	0.19
GOAL	0.85	0.28	0.23	0.88	0.67*	[0.55; 0.78]	0.40	0.07
No-Apnea	0.76	0.42	0.25	0.88	0.70**	[0.58; 0.81]	0.49	0.10

* *p* < 0.05; ** *p* < 0.001; _ *p* > 0.8

*Se *sensibility, *Sp *specificity, *PPV *predictive positive value, *NPV *predictive negative value, *AUC *area under the curve, *CI *Confidence Interval

The performance of the different items from the various questionnaires is reported in Appendix 1 (Table 5, 6, and 7).

Threshold optimization using the Youden index identified higher cut-offs than the standard ones for some scores: No-Apnea = 6 (vs. 4), NoSAS = 10 (vs. 8), and GOAL = 3 (vs. 2). These adjustments slightly improved observed agreement while maintaining a good sensitivity/specificity trade-off (Appendix Table 8).

Hypertension, obesity, and age were the comorbidities associated with OSAHS (Tables 3 and 4). In multivariate analysis for moderate-to-severe OSAHS, adjusted for nine variables (age, BMI, neck circumference, male sex, loud snoring, frequent apneas, frequent daytime fatigue, hypertension, diabetes), age (OR 1.06 [1.02–1.10], *p* = 0.003), BMI (OR 1.10 [1.01–1.20], *p* = 0.045), and neck circumference (OR 1.27 [1.07–1.51], *p* = 0.007) were the only independent risk factors.

Discriminant analysis of individual questionnaire items (Appendices Table 5, 6 and 7).

Loud snoring was associated with both mild and severe OSAHS. Frequent snoring was associated only with mild OSAHS. Category 1 of the Berlin questionnaire, which combines several snoring-related questions, was associated only with mild OSAHS. Category 3 of the Berlin questionnaire, which combines hypertension and BMI, was associated with all OSAHS severity levels.

**Table 3:** Univariate analysis of comorbidities associated with OSAHS

	AHI ≥ 5	AHI < 5	*p*
Male sex	41 (37.6%)	9 (36%)	1.000
Hypertension	55 (50.5%)	5 (20%)	**0.007**
Diabetes	23 (21.1%)	2 (8%)	0.162
Dyslipidemia	10 (9.2%)	1 (4%)	0.689
Allergies	31 (28.4%)	8 (32%)	0.808
Rhinitis	10 (9.2%)	3 (12%)	0.709
Sinusitis	13 (11.9%)	3 (12%)	1.000
Asthma	27 (24.8%)	6 (24%)	1.000
	AHI ≥ 15	AHI < 15	p
Male sex	25 (44.6%)	25 (32.1%)	0.151
Hypertension	30 (53.6%)	30 (38.5%)	0.113
Diabetes	11 (19.6%)	14 (17.9%)	0.825
Dyslipidemia	6 (10.7%)	5 (6.4%)	0.525
Allergies	14 (25%)	25 (32.1%)	0.443
Rhinitis	4 (7.1%)	9 (11.5%)	0.557
Sinusitis	6 (10.7%)	10 (12.8%)	0.792
Asthma	16 (28.6%)	17 (21.8%)	0.416
	AHI ≥ 30	AHI < 30	p
Male sex	14 (51.9%)	36 (33.6%)	0.118
Hypertension	15 (55.6%)	45 (42.1%)	0.279
Diabetes	5 (18.5%)	20 (18.7%)	1.000
Dyslipidemia	2 (7.4%)	9 (8.4%)	1.000
Allergies	4 (14.8%)	35 (32.7%)	0.096
Rhinitis	2 (7.4%)	11 (10.3%)	1.000
Sinusitis	2 (7.4%)	14 (13.1%)	0.525
Asthma	7 (25.9%)	26 (24.3%)	1.000

**Table 4:** Univariat e analysis of anthropometric variables associated with OSAHS

	IAH ≥ 5	IAH < 5	*p*
Age	58.8 ± 14.2	49.8 ± 15.2	0.012
Weight	89.1 ± 23.7	83.8 ± 22.2	0.318
Height	169.6 ± 9.9	171.2 ± 9.8	0.476
BMI	30.9 ± 7.5	28.6 ±7.2	0.182
Neck circumference	39.8 ± 4.2	37.8 ± 4.1	**0.046**
Waist circumference	107.4 ± 16.5	99.2 ± 14.2	**0.034**
	IAH ≥ 15	IAH < 15	p
Age	59.7 ± 15.6	55.2 ± 13.8	0.089
Weight	96.2 ± 26.7	82.1 ± 18.7	**0.001**
Height	169.6 ± 11.9	170.2 ± 8.1	0.783
BMI	33.3 ± 8.3	28.4 ± 6.1	**< 0.001**
Neck circumference	41.5 ± 4.1	38.0 ± 3.8	**< 0.001**
Waist circumference	114.7 ± 16.1	99.7 ± 13.5	**< 0.001**
	IAH ≥ 30	IAH < 30	p
Age	59.1 ± 17.2	56.6 ± 14.1	0.489
Weight	104.1 ± 31.9	84.0 ± 18.7	**0.004**
Height	171.9 ± 13.3	169.5 ± 8.9	0.381
BMI	35.1 ± 9.7	29.3 ± 6.3	**0.007**
Neck circumference	42.1 ± 5.0	38.8 ± 3.8	**0.004**
Waist circumference	116.2 ± 15.1	103.3 ± 15.7	**0.001**

Mean ± SD

## Discussion

Our study shows that, in a Martinique population referred for suspected OSAHS, the diagnostic performances of the six evaluated screening questionnaires ranked as follows: NoSAS, No-Apnea, STOP-BANG, GOAL, Berlin Questionnaire, and Epworth Sleepiness Scale.

In particular, the Epworth scale performed no better than chance, highlighting its limited relevance as a screening tool despite its widespread use in practice. The DeLong test comparing area under the curve (AUC) values pairwise confirmed this ranking statistically.

For the best-performing questionnaires, AUCs exceeded 0.70, supporting their potential utility for screening. However, Cohen’s kappa coefficients remained low, reflecting limited diagnostic agreement. Several factors may explain this:

- a high OSAHS prevalence in our sample (referral bias).
- strong correlations between questionnaires, reducing their ability to distinguish patients.
- suboptimal thresholds for our population.

Optimizing thresholds using the Youden index identified higher cut-offs than standard values: 6 (vs. 4) for No-Apnea, 10 (vs. 8) for NoSAS, and 3 (vs. 2) for GOAL. These adjustments substantially improved kappa values while maintaining a good sensitivity/specificity balance. The other questionnaires retained their usual thresholds.

Our findings are broadly consistent with international data. NoSAS and STOP-BANG are regularly reported as the most accurate screening tools in the literature [13, 14]. In a meta-analysis, Chiu HY et al. reported the highest sensitivities for the STOP-BANG and STOP questionnaires regardless of OSAHS severity. The Berlin Questionnaire showed better sensitivity for severe OSAHS. Specificities were generally low but highest for the Epworth Sleepiness Scale and Berlin questionnaire [21]. NoSAS, No-Apnea, and GOAL were not assessed in that analysis.

In a study conducted in China, Qing SM et al. found that NoSAS and STOP-BANG outperformed STOP, Berlin, and Epworth Sleepiness Scale [22]. In patients with cerebral infarction, Chen R et al. reported acceptable performance for NoSAS, STOP-BANG, STOP, and Berlin, but poor performance for No-Apnea and Epworth Sleepiness Scale [23]. Among elderly Chinese patients, the Berlin questionnaire showed acceptable performance compared with NoSAS, STOP-BANG, GOAL, and Epworth Sleepiness Scale [24]. A recent meta-analysis by Li X et al. recommended STOP for general population screening due to its good sensitivity, and Epworth Sleepiness Scale for use by healthcare professionals due to its better specificity [25]. These findings highlight the heterogeneity of screening questionnaire performance across studies. Studies in United States African American populations have also emphasized the value of scores based on objective criteria (neck circumference, BMI, hypertension) rather than subjective ones [15–17]. However, several works suggest these tools require recalibration in Black populations: for example, the Berlin questionnaire showed modest sensitivity (≈ 46% for AHI ≥ 15) in a predominantly Black sample, and while STOP-BANG was highly sensitive, its specificity was generally low [19]. Dedicated approaches, such as the model developed in the Jackson Heart Sleep Study, aim to incorporate the anthropometric and clinical specificities of these populations [20]. Our finding that local threshold optimization improved observed agreement (Appendix Table 8) is consistent with this approach of adapting tools to the ethnic and sociocultural context.

Regarding anthropometric factors, univariate analyses showed that age was associated with mild OSAHS, while waist circumference, neck circumference, BMI, and weight were associated with moderate-to-severe forms. In our multivariate model, age, BMI, and neck circumference remained independently associated with moderate-to-severe OSAHS after adjustment. The clinical profile of mild OSAHS (predominantly female, older, hypertensive, and obese) matched the population with the highest prevalence in Martinique. Severe forms resembled the typical obese male phenotype commonly described in the literature.

## Strengths and limitations

To our knowledge, this is the first study to evaluate screening questionnaires for OSAHS in an Afro-Caribbean population. It contributes to a better characterization of this specific population, which is probably heavily affected by OSAHS, similarly to obesity and cardiovascular diseases. As patient-reported outcome measures, questionnaires such as the Epworth Sleepiness Scale and the Berlin questionnaire may be sensitive to cross-cultural and linguistic differences that affect the measurement of the underlying constructs. Some items refer to situations that may be less frequent or perceived differently depending on daily activities and lifestyle. For example, ESS items involving situations such as prolonged car driving may be less representative in settings with shorter travel times or different leisure habits, potentially influencing symptom reporting. These contextual factors may contribute to measurement variability and should be considered when interpreting PRO-based screening tools in culturally distinct populations. Despite these limitations, questionnaire completion was generally feasible in routine practice, with limited missing data, supporting their acceptability for primary care screening.

However, several limitations should be acknowledged:

- **Selection bias**: Most included patients were referred for suspected OSAHS, which likely overestimates the true prevalence. Thus, in a general population the positive predictive value of these questionnaires would likely decrease, while specificity might increase, potentially altering their clinical utility compared with a referred high-risk population.
- **Use of a quasi–gold standard**: Although respiratory polygraphy is acceptable in routine practice—especially in high-prevalence settings such as ours, it has limitations compared to polysomnography, notably for detecting microarousals, assessing sleep fragmentation, and measuring total sleep time. These limitations may introduce misclassification bias, especially for mild OSAHS, leading to underestimation of disease severity and potentially reduced sensitivity.
- **Missing data**: Some variables were poorly reported, especially in the Epworth Sleepiness Scale and Berlin Questionnaire, which may have weakened their performance. Moreover, these tools require cross-cultural adaptation, as some items describe situations that are uncommon in our patients’ daily lives.
- Validation: The MASOS score was derived within the same dataset and was neither internally validated nor calibrated, which may result in optimistic performance estimates. The threshold optimization was performed within the same dataset, which may have led to overfitting and optimistic estimates of diagnostic performance.

### Perspectives

The NoSAS score appears to be the most suitable tool for primary care in Martinique. However, the AUC of the full multivariable model, which included clinical covariates, was clearly higher than that of the NoSAS score alone (0.81 vs. 0.73). Although this difference was not statistically significant according to the DeLong test, it suggests that simply adjusting the threshold may not be sufficient and that a specifically calibrated tool would be more appropriate. We refined the model to retain only three covariates—age, BMI, and neck circumference—while maintaining an AUC of 0.81. This model allowed us to develop the MASOS score, which achieved an AUC of 0.79.

The MASOS score (Age ≥ 70 years + 2; BMI ≥ 30 kg/m² +1; Neck circumference ≥ 39 cm + 1; with a threshold of > = 2) seems better for our population than other scores with an AUC of 0.79 but still need to be validated. 

The MASOS score was derived from a reduced multivariable model including age. body mass index (BMI). and neck circumference. A cut-off ≥ 2 points yielded an AUC of 0.788. with a sensitivity of 0.81. specificity of 0.69. PPV of 0.66. and NPV of 0.83 (Table 10). A prospective study in the general population of Martinique, using polysomnography as the gold standard, would be needed to confirm the usefulness of this local model and the MASOS score (Appendix Table 9) for screening in primary care.

## Conclusion

OSAHS is a major public health issue, with a potentially dramatic increase in prevalence in the coming decades. In the context of limited medical resources, using screening tools can improve the efficiency of patient management. In our setting, the best-performing questionnaires were the NoSAS and No-Apnea scores. However, they require optimization, formal evaluation, and possibly cross-cultural adaptation.

Based on a multivariable model combining age, BMI, and neck circumference, we developed the MASOS score (Age ≥ 70 years + 2; BMI ≥ 30 kg/m² +1; neck circumference ≥ 39 cm + 1; threshold ≥ 2), which showed a higher discriminative performance (AUC = 0.79). This score still requires validation.

## Data Availability

The datasets generated and/or analyzed during the current study are available from the corresponding author on reasonable request.

## Appendix

### Analysis of questionnaire items associated with OSAHS

**Table 5:** Analysis of questionnaire items associated with mild OSAHS (AHI ≥ 5)

	AHI ≥ 5 *n* (%) *N* = 109	AHI < 5 *n* (%) *N* = 25	OR [95% CI]	AUC	*p*	
Do you snore?	87 (79.8%)	16 (64%)	2.22 [0.87-5.70]	0.58	0.115	
Loud snoring	54 (49.5%)	4 (16%)	**5.15 [1.66–16.01]**	**0.67**	**0.003**	
Frequent snoring > 1d/2	71 (65.1%)	10 (40%)	**2.96 [1.21–7.24]**	**0.63**	**0.022**	
Snoring disturbing others	54 (49.5%)	9 (36%)	1.81 [0.74–4.46]	0.57	0.266	
Witnessed apnea	40 (36.7%)	8 (32%)	1.23 [0.49–3.11]	0.52_	0.818	
Frequent apnea > 1d/2	28 (25.7%)	2 (1.5%)	4.08 [0.90–18.41]	0.59	0.064	
Daytime fatigue	72 (66.1%)	16 (11.9%)	1.09 [0.44–2.71]	0.51_	0.820	
Frequent morning fatigue > 1d/2	56 (51.4%)	12 (9.1%)	1.19 [0.50–2.84]	0.52_	0.825	
Frequent daytime fatigue > 1d/2	65 (59.6%)	13 (9.8%)	1.43 [0.60–3.43]	0.54	0.500	
Falling asleep while driving once	23 (21.1%)	5 (3.8%)	1.10 [0.37–3.24]	0.51_	1.000	
Falling asleep while driving > 1d/2	6 (5.5%)	3 (12%)	0.44 [0.10–1.88]	0.47	0.370	
Berlin category 1	77 (70.8%)	12 (48%)	**2.78 [1.14–6.78]**	**0.62**	**0.032**	
Berlin category 2	55 (50.5%)	11 (44%)	1.35 [0.56–3.23]	0.54	0.657	
Berlin category 3	85 (78%)	12 (48%)	**3.70 [1.47–9.30]**	**0.64**	**0.009**	
	Median [IQ]	Median [IQ]		AUC		p
Epworth: Sitting and reading	1 [0; 2]	1 [0; 2]		0.49_		0.826
Epworth: Watching television	2 [1; 2]	2 [0.25; 2]		0.51_		0.873
Epworth: Inactive in a public place (theater. cinema. meeting)	0 [0; 2]	0.5 [0; 1]		0.54		0.550
Epworth: Passenger in a car driving for 1 h	0 [0; 2]	0 [0; 1]		0.57_		0.330
Epworth: Lying down to rest in the afternoon	3 [1; 3]	2.5 [1.25; 3]		0.51		0.861
Epworth: Sitting and talking to someone	0 [0; 0]	0 [0; 0]		0.53		0.707
Epworth: Sitting quietly after a meal without alcohol	1 [0; 2]	1 [0.25; 2]		0.50_		0.968
Epworth: In a car stopped for a few minutes	0 [0; 1]	0 [0; 0]		0.59		0.165

**Table 6:** Analysis of questionnaire items associated with moderate OSAHS (AHI ≥ 15)

	AHI ≥ 15 *n* (%) *N* = 56	AHI < 15 *n* (%) *N* = 78	OR [95% CI]	AUC	*p*
Do you snore?	43 (76.8%)	60 (76.9%)	0.99 [0.44–2.24]	0.50_	1.000
Loud snoring	27 (48.2%)	31 (39.7%)	1.41 [0.71–2.82]	0.54	0.378
Frequent snoring > 1d/2	37 (66.1%)	44 (56.4%)	1.42 [0.69–2.90]	0.54	0.370
Snoring disturbing others	30 (53.6%)	33 (42.3%)	1.50 [0.75–3.01]	0.55	0.292
Witnessed apnea	20 (35.7%)	28 (35.9%)	0.99 [0.48–2.03]	0.50_	1.000
Frequent apnea > 1d/2	15 (26.8%)	15 (19.2%)	1.49 [0.66–3.37]	0.54	0.402
Daytime fatigue	36 (64.3%)	52 (66.7%)	0.90 [0.44–1.85]	0.49_	0.854
Frequent morning fatigue > 1d/2	29 (51.8%)	39 (50%)	1.02 [0.51–2.03]	0.50_	1.000
Frequent daytime fatigue > 1d/2	33 (58.9%)	45 (57.7%)	0.99 [0.49–1.99]	0.50_	1.000
Falling asleep while driving once	11 (19.6%)	17 (21.8%)	0.85 [0.36–1.99]	0.49_	0.830
Falling asleep while driving > 1d/2	4 (7.1%)	5 (6.4%)	1.09 [0.28–4.27]	0.50_	1.000
Berlin category 1	39 (69.6%)	50 (64.1%)	1.19 [0.57–2.50]	0.52	0.709
Berlin category 2	27 (48.2%)	39 (50%)	0.88 [0.44–1.76]	0.48_	0.860
Berlin category 3	49 (87.5%)	48 (51.5%)	**4.08 [1.63–10.24]**	**0.62***	**0.002**
	Median [IQ]	Median [IQ]		AUC	p-value
Epworth: Sitting and reading	1 [0; 2]	1 [0; 2]		0.49_	0.886
Epworth: Watching television	2 [1; 3]	2 [1; 2]		0.52	0.757
Epworth: Inactive in a public place (theater. cinema. meeting)	0 [0; 1.25]	1 [0; 1]		0.50_	0.978
Epworth: Passenger in a car driving for 1 h	0 [0; 2]	0 [0; 1]		0.53	0.527
Epworth: Lying down to rest in the afternoon	3 [2; 3]	2 [1; 3]		0.54	0.502
Epworth: Sitting and talking to someone	0 [0; 0]	0 [0; 0]		0.50_	0.957
Epworth: Sitting quietly after a meal without alcohol	1.5 [0; 2.25]	1 [0; 2]		0.52	0.757
Epworth: In a car stopped for a few minutes	0 [0; 0.5]	0 [0; 1]		0.49_	0.803

**Table 7:** Analysis of questionnaire items associated with severe OSAHS (AHI ≥ 30)

	AHI ≥ 30 *n* (%) *N* = 27	AHI < 30 *n* (%) *N* = 107	OR [95% CI]	AUC	*p*
Do you snore?	22 (81.5%)	81 (75.7%)	1.41 [0.49–4.10]	0.53	0.617
Loud snoring	17 (63%)	41 (38.3%)	**2.74 [1.14–6.55]**	**0.62***	**0.029**
Frequent snoring > 1d/2	19 (70.4%)	62 (57.9%)	1.65 [0.66–4.10]	0.56	0.376
Snoring disturbing others	13 (48.1%)	50 (46.7%)	1.02 [0.44–2.38]	0.50_	1.000
Witnessed apnea	11 (40.7%)	37 (34.6%)	1.30 [0.55–3.09]	0.53	0.654
Frequent apnea > 1d/2	9 (33.3%)	21 (19.6%)	2.00 [0.79–5.08]	0.57	0.196
Daytime fatigue	18 (66.7%)	70 (65.4%)	1.06 [0.43–2.58]	0.51_	1.000
Frequent morning fatigue > 1d/2	14 (51.9%)	54 (50.5%)	1.02 [0.44–2.37]	0.50_	1.000
Frequent daytime fatigue > 1d/2	14 (51.9%)	64 (59.8%)	0.69 [0.29–1.62]	0.45	0.511
Falling asleep while driving once	5 (18.5%)	23 (21.5%)	0.81 [0.28–2.38]	0.48	0.797
Falling asleep while driving > 1d/2	2 (7.4%)	7 (6.5%)	1.12 [0.22–5.73]	0.50_	1.000
Berlin category 1	21 (77.8%)	68 (63.6%)	1.90 [0.71–5.13]	0.57	0.253
Berlin category 2	12 (44.4%)	54 (50.5%)	0.76 [0.32–1.77]	0.47	0.667
Berlin category 3	25 (92.6%)	81 (67.3%)	**5.73 [1.28–25.63]**	**0.62***	**0.013**
	Median [IQR]	Median [IQR]		AUC	p
Epworth: Sitting and reading	1.5 [0; 2]	1 [0; 2]		0.53	0.611
Epworth: Watching television	2 [1; 3]	2 [1; 2]		0.56	0.392
Epworth: Inactive in a public place (theater. cinema. meeting)	0 [0; 2]	0.5 [0; 1]		0.52	0.727
Epworth: Passenger in a car driving for 1 h	0 [0; 1]	0 [0; 1]		0.47	0.628
Epworth: Lying down to rest in the afternoon	3 [2; 3]	3 [1; 3]		0.54	0.505
Epworth: Sitting and talking to someone	0 [0; 0]	0 [0; 0]		0.52	0.777
Epworth: Sitting quietly after a meal without alcohol	2 [0; 3]	1 [0; 2]		0.53	0.669
Epworth: In a car stopped for a few minutes	0 [0; 1]	0 [0; 1]		0.51_	0.887

**Table 8:** Threshold optimization of screening questionnaires using the Youden index

	Best threshold	Se	Sp	Agreement	Kappa
AH I ≥ 5					
Epworth	17	0.11	1.00	0.27	0.04
Berlin	2	0.71	0.58	0.68	0.22
STOP-BANG	3	0.74	0.68	0.73	0.31
NoSAS	9	0.67	0.77	0.69	0.29
GOAL	2	0.81	0.54	0.76	0.31
No-Apnea	6	0.46	0.91	0.54	0.19
AHI ≥ 15					
Epworth	16	0.18	0.91	0.60	0.10
Berlin	2	0.71	0.39	0.53	0.10
STOP-BANG	3	0.79	0.43	0.58	0.21
NoSAS	10	0.69	0.75	0.72	0.43
GOAL	3	0.41	0.85	0.66	0.28
No-Apnea	6	0.65	0.81	0.74	0.46
AHI ≥ 30					
Epworth	15	0.26	0.86	0.73	0.13
Berlin	2	0.78	0.38	0.46	0.09
STOP-BANG	6	0.30	0.94	0.80	0.28
NoSAS	11	0.78	0.66	0.69	0.32
GOAL	3	0.52	0.81	0.75	0.30
No-Apnea	6	0.68	0.69	0.69	0.27

**Table 9:** The MASOS score derived from the reduced multivariable model

Variable	Points
Age ≥ 70 years	2
BMI ≥ 30 kg/m²	1
Neck circumference ≥ 39 cm	1

The MASOS score was derived from a reduced multivariable model including age. body mass index (BMI). and neck circumference. A cut-off ≥ 2 points yielded an AUC of 0.788. with a sensitivity of 0.81. specificity of 0.69. PPV of 0.66. and NPV of 0.83 (Table 10).

**Table 10:** Logistic regression model for independent risk factor for moderate-to-severe OSAHS

Variable	β (Coefficient)	OR	IC 95%	*P*
Age (year)	0.0539	1.055	[1.019; 1.093]	0.0021
BMI (kg/m²)	0.1030	1.108	[1.028; 1.194]	0.0074
Neck circumference (cm)	0.1938	1.214	[1.074; 1.372]	0.0020
Constant	−11.873	-	-	< 0.001
